# Medical Convention

**Published:** 1866-08

**Authors:** A. H. Thompson, Geo. H. Scott

**Affiliations:** Pres’t.; Sec’y.


### MEDICAL CONVENTION.

Pursuant to previous notice, physicians from the counties of Bureau, Henry, Knox, and Stark met at Kewanee, May 22d, 1866, to organize a District Medical Association.

On motion, H. S. Hurd, M.D., of Galesburg, was called to the Chair, and Geo. H. Scott, M.D., chosen Secretary.

On motion, proceeded to the organization of a District Medical Association.

On motion, a committee of three, consisting of Drs. N. Holton, of Buda, Jas. M. Morse, of Galesburg, and J. C. Smiley, of Kewanee, were appointed, to present a constitution and by-laws for said association.

The committee submitted the following Constitution and By-Laws which, on motion, (the committee being discharged,) were approved and adopted, article by article:—

CONSTITUTION.

Art. 1. This Association shall be known and designated as the Military Tract Medical Association.

Art. 2. This Association is to embrace the members of the regular medical profession, who are in good standing in the profession, in the counties of Bureau, Henry, Stark, Knox, and Warren.

Art. 3. The physicians present at the adoption of this Constitution, may be members of the Association.

Art. 4. Any regular graduate of an orthodox school, or any physician passing a satisfactory examination before the Board of Censors, may become a member by a vote of the Association.

Art. 5. The officers of this Association shall consist of a President, Vice-President, and Secretary, who shall act as Treasurer, and three Censors; to be elected annually, by ballot, by a majority vote of all the members present.

Art. 6. This Association shall be governed by the Code of Ethics adopted by the American Medical Association.

Art. 7. This Constitution may be altered or amended by a vote of two-thirds of the members present at any regular meeting.

BY-LAWS.

Art. 1. The President shall appoint, at each regular meeting, two members to prepare and deliver, at the next regular meeting, an essay on some medical subject, or report a case.

Art. 2. The report of cases and essays made before the Association at its regular meetings, shall become the property of this Association.

Art. 3. Any member who has conducted himself properly during his membership, shall, if he require it, receive a certificate of character, signed by the President and Secretary.

Art. 4. The meetings of this Association shall be held twice a-year, in the months of May and December.

On motion, the Association proceeded to the election of officers, when the following were duly elected:—

*For President.*—A. H. Thomson, M.D., Princeton.

*For Vice-President.*—H. Nance, M.D., Kewanee.

*For Secretary and Treasurer.*—Geo. H. Scott, M.D., Kewanee.

*For Censors.*—Drs. N. Holton, Buda; Jno. M. Morse, Galesburg; and V. C. Secord, Galva.

*For Delegates to the State Medical Convention.*—Drs. H. Nance, Kewanee; S. P. Breed, Princeton; Jno. M. Morse, Galesburg; W. C. Brown, Geneseo; and A. D. Babcock, Galva.

Moved and adopted, that the initiation fee shall be $1.00.

Moved and adopted, that the Secretary purchase a blank book, at the expense of six or eight dollars, in which to record the proceedings of the Association.

Dr. A. H. Thomson offered the following, as the order of business. Approved:—

ORDER OF BUSINESS.

1st. Reading the minutes of last meeting.

2d. Report of Censors on credentials for admission.

3d. Election of officers at annual meeting.

4th. Reading of essays, and discussion.

5th. Report of cases and presenting morbid specimens.

6th. Miscellaneous business.

On motion, it was decided that our next meeting be held at Galesburg, on the second Tuesday in December, 1866.

Moved by Dr. Holton, and adopted, that one from each county be appointed a committee to report on the endemic and epidemic diseases of the county.

The following physicians were appointed:—Drs. N. Holton, Buda; Jno. M. Morse, Galesburg; H. Nance, Kewanee; and E. K. Boardman, Elmira.

Moved by Dr. Babcock, and adopted, that the Secretary notify each member, by circular, one month before the ensuing meeting.

A vote of thanks was passed to the medical brotherhood of Kewanee, for the hospitable entertainments extended to the members of the Association; also to the trustees of the Congregational Church, for the use of their edifice.

The following is the list of members:—

Drs. H. S. Hurd, Galesburg; Geo. H. Scott, Kewanee; A. H. Thompson, Princeton; J. C. Secord, Galva; Jno. M. Morse, Galesburg; A. C. Babcock and A. D. Babcock, Galva; N. Holton, Buda; Geo. W. Crossley and S. P. Breed, Princeton; G. H. Vance, Victoria; H. Nance, Kewanee; S. T. Hume, Geneseo; C. M. Clark, Galva; W. C. Brown, Geneseo; W. H. Day, Kewanee; E. K. Boardman, Elmira; J. C. Smiley, Kewanee.

On motion, the Secretary was instructed to have the proceedings of the Association published in the papers in this District; also in the medical journals of the State.

On motion, the Association adjourned, to meet in Galesburg on the second Tuesday in December.

A. H. THOMPSON, M.D., *Pres't.*

GEO. H. SCOTT, M.D., *Sec'y.*

